# The Advisory Committee on Immunization Practices’ Interim Recommendations for Additional Primary and Booster Doses of COVID-19 Vaccines — United States, 2021

**DOI:** 10.15585/mmwr.mm7044e2

**Published:** 2021-11-05

**Authors:** Sarah Mbaeyi, Sara E. Oliver, Jennifer P. Collins, Monica Godfrey, Neela D. Goswami, Stephen C. Hadler, Jefferson Jones, Heidi Moline, Danielle Moulia, Sujan Reddy, Kristine Schmit, Megan Wallace, Mary Chamberland, Doug Campos-Outcalt, Rebecca L. Morgan, Beth P. Bell, Oliver Brooks, Camille Kotton, H. Keipp Talbot, Grace Lee, Matthew F. Daley, Kathleen Dooling

**Affiliations:** ^1^CDC COVID-19 Response Team; ^2^Epidemic Intelligence Service, CDC; ^3^University of Arizona, College of Medicine, Phoenix, Arizona; ^4^Department of Health Research Methods, Evidence and Impact, Hamilton, Ontario; ^5^University of Washington, Seattle, Washington; ^6^Watts HealthCare Corporation, Los Angeles, California; ^7^Harvard Medical School, Boston, Massachusetts; ^8^Vanderbilt University School of Medicine, Nashville, Tennessee; ^9^Stanford University School of Medicine, Stanford, California; ^10^Institute for Health Research, Kaiser Permanente Colorado, Denver, Colorado.

Three COVID-19 vaccines are currently approved under a Biologics License Application (BLA) or authorized under an Emergency Use Authorization (EUA) by the Food and Drug Administration (FDA) and recommended for primary vaccination by the Advisory Committee on Immunization Practices (ACIP) in the United States: the 2-dose mRNA-based Pfizer-BioNTech/Comirnaty and Moderna COVID-19 vaccines and the single-dose adenovirus vector-based Janssen (Johnson & Johnson) COVID-19 vaccine (*1*,*2*) (Box 1). In August 2021, FDA amended the EUAs for the two mRNA COVID-19 vaccines to allow for an additional primary dose in certain immunocompromised recipients of an initial mRNA COVID-19 vaccination series (*1*). During September–October 2021, FDA amended the EUAs to allow for a COVID-19 vaccine booster dose following a primary mRNA COVID-19 vaccination series in certain recipients aged ≥18 years who are at increased risk for serious complications of COVID-19 or exposure to SARS-CoV-2 (the virus that causes COVID-19), as well as in recipients aged ≥18 years of Janssen COVID-19 vaccine (*1*) (Table). For the purposes of these recommendations, an additional primary (hereafter additional) dose refers to a dose of vaccine administered to persons who likely did not mount a protective immune response after initial vaccination. A booster dose refers to a dose of vaccine administered to enhance or restore protection by the primary vaccination, which might have waned over time. Health care professionals play a critical role in COVID-19 vaccination efforts, including for primary, additional, and booster vaccination, particularly to protect patients who are at increased risk for severe illness and death.

BOX 1 **Timeline of COVID-19 vaccine authorizations, approvals, and vaccine recommendations — United States, December 2020–October 2021**

**December 2020**

- FDA authorizes and ACIP recommends Pfizer-BioNTech COVID-19 vaccine as a 2-dose primary series in persons aged ≥16 years.
- FDA authorizes and ACIP recommends Moderna COVID-19 vaccine as a 2-dose primary series in persons aged ≥18 years.
- ACIP issues interim recommendations for allocating initial supplies of COVID-19 vaccine, starting with long-term care facility residents and health care personnel, followed by other groups at risk.

**February 2021**

- FDA authorizes and ACIP recommends Janssen (Johnson & Johnson) COVID-19 vaccine as a single dose in persons aged ≥18 years.

**April 2021**

- CDC and FDA recommend pausing use of Janssen COVID-19 vaccine after reports of thrombosis with thrombocytopenia syndrome (TTS) among vaccine recipients. Ten days later, ACIP concludes that the benefits of resuming Janssen COVID-19 vaccination outweighs the risks and reaffirms its interim recommendations under FDA’s Emergency Use Authorization, which includes a new warning for TTS among women aged 18–49 years.

**May 2021**

- FDA authorizes and ACIP recommends Pfizer-BioNTech COVID-19 vaccine as a 2-dose primary series in persons aged 12–15 years.

**June 2021**

- After reports of myocarditis among mRNA COVID-19 vaccine recipients, particularly males aged 12–29 years, ACIP concludes that the benefits of COVID-19 vaccination outweigh the risks of myocarditis after vaccination.

**July 2021**

- After reports of Guillain-Barré syndrome (GBS) in Janssen COVID-19 vaccine recipients, ACIP reviews updated benefit-risk analyses for all COVID-19 vaccines and concludes that the benefits of vaccination outweigh the risks of GBS and TTS (Janssen COVID-19 vaccine) and myocarditis (mRNA COVID-19 vaccines).

**August 2021**

- FDA grants full approval and ACIP revises its interim recommendation to a standard recommendation for Pfizer-BioNTech COVID-19 vaccine (Comirnaty) for persons aged ≥16 years.
- FDA authorizes and ACIP recommends an additional primary dose of Pfizer-BioNTech COVID-19 (for persons aged ≥12 years) and Moderna COVID-19 vaccine (for persons aged ≥18 years) in certain immunocompromised persons ≥28 days after completion of the second dose in the initial primary series.

**September 2021**

- FDA authorizes a single Pfizer-BioNTech COVID-19 vaccine booster dose in certain persons aged ≥18 years at increased risk for serious complications of COVID-19, including severe COVID-19, who completed a Pfizer-BioNTech COVID-19 vaccine primary series ≥6 months ago.
- ACIP recommends that persons aged ≥65 years, residents aged ≥18 years in long-term care settings, and persons aged 50–64 years with certain underlying medical conditions should receive a booster dose and that persons aged 18–49 years with certain underlying medical conditions may receive a single Pfizer-BioNTech COVID-19 vaccine booster dose based on individual benefits and risks.
- CDC further recommends that persons aged 18–64 years at increased risk for SARS-CoV-2 exposure and transmission because of occupational or institutional setting may receive a single Pfizer-BioNTech COVID-19 vaccine booster dose based on individual benefits and risks. This recommendation is replaced by ACIP’s broader recommendation in October 2021 for COVID-19 vaccine booster doses in persons who received an mRNA primary series (described below).

**October 2021**

- FDA authorizes a single Moderna COVID-19 vaccine booster dose (50 *μ*g) in persons aged ≥18 years at increased risk for serious complications of COVID-19 or frequent institutional or occupational exposure to SARS-CoV-2 who completed a Moderna COVID-19 vaccine primary series ≥6 months ago.
- FDA authorizes a single Janssen COVID-19 vaccine booster dose in persons aged ≥18 years who received a Janssen COVID-19 vaccine dose ≥2 months ago.
- FDA authorizes that Pfizer-BioNTech, Moderna, and Janssen COVID-19 vaccines may be administered as a heterologous booster dose after completion of primary vaccination with another COVID-19 vaccine.
- ACIP recommends that persons aged ≥65 years, residents aged ≥18 years in long-term care settings, and persons aged 50–64 years with certain underlying medical conditions who received an mRNA COVID-19 vaccine primary series should receive a single COVID-19 vaccine booster dose ≥6 months later; persons aged 18–49 years with certain underlying medical conditions and persons aged 18–64 years at increased risk for SARS-CoV-2 exposure and transmission because of occupational or institutional setting may receive a COVID-19 booster dose based on individual benefits and risks. This recommendation replaces the September 23, 2021, ACIP recommendation for Pfizer-BioNTech COVID-19 vaccine booster doses.
- ACIP recommends that persons aged ≥18 years who received Janssen COVID-19 vaccine should receive a single COVID-19 vaccine booster dose ≥2 months later.

**Abbreviations:** ACIP = Advisory Committee on Immunization Practices; FDA = Food and Drug Administration.

**TABLE:** COVID-19 vaccines approved or authorized for emergency use by the Food and Drug Administration for persons aged ≥12 years — United States, October 2021*

COVID-19 vaccine manufacturer	Primary and additional primary doses					Booster dose^†^	
	Age, yrs	Dose (volume)	No. of doses (interval between doses)	Additional primary dose in immunocompromised persons (interval since 2nd dose)	Interval between last primary (including additional) dose to booster dose	Dose (volume)	No. of doses
**Pfizer-BioNTech (Comirnaty)**	12–17	30 *μ*g (0.3 ml)	2 (21 days)	1 (≥28 days)	NA	NA	NA
	≥18^§^	30 *μ*g (0.3 ml)	2 (21 days)	1 (≥28 days)	≥6 months	30 *μ*g (0.3 ml)	1
**Moderna**	≥18^§^	100 *μ*g (0.5 ml)	2 (28 days)	1 (≥28 days)	≥6 months	50 *μ*g (0.25 ml)	1
**Janssen (Johnson & Johnson)**	≥18	5 × 10^10^ VP (0.5 ml)	1 (NA)	NA	≥2 months	5 × 10^10^ VP (0.5 ml)	1

**Abbreviations:** NA = not applicable; VP = viral particles.

* As of October 29, 2021. Food and Drug Administration package inserts or fact sheets provide updated information at https://www.fda.gov/emergency-preparedness-and-response/coronavirus-disease-2019-covid-19/covid-19-vaccines. Route of administration is intramuscular for the currently approved or authorized COVID-19 vaccines.

^†^ The Advisory Committee on Immunization Practices recommends that any of the FDA-approved or authorized COVID-19 vaccines (Pfizer-BioNTech, Moderna, or Janssen) may be used for booster vaccination, regardless of the vaccine product used for primary vaccination. When a heterologous (mix-and-match) booster dose is administered, the eligible population and dosing intervals are those of the vaccine used for primary vaccination. For example, a recipient of Janssen COVID-19 vaccine may receive a Pfizer-BioNTech, Moderna, or Janssen COVID-19 vaccine booster dose ≥2 months after their primary dose. A recipient of an mRNA COVID-19 primary series who is in a risk group recommended to receive a booster dose may receive a Pfizer-BioNTech, Moderna, or Janssen COVID-19 vaccine booster dose ≥6 months after completion of the primary series. Additional information on groups recommended for an additional primary or booster dose of COVID-19 vaccine is provided at https://www.cdc.gov/vaccines/covid-19/clinical-considerations/covid-19-vaccines-us.html.

^§^ Minimum authorized age group; see Advisory Committee on Immunization Practices’ recommendations for groups recommended to receive a booster dose.

After the EUA amendments, ACIP and CDC issued interim recommendations for vaccine use*^,^^†^^,^^§^ (*2*). Moderately to severely immunocompromised persons aged ≥12 years (Pfizer-BioNTech recipients) or ≥18 years (Moderna recipients) should receive an additional homologous dose of mRNA COVID-19 vaccine (i.e., the same vaccine product that was administered for the primary series) ≥28 days after receipt of the second dose. Regarding booster dose recommendations, recipients of a primary mRNA COVID-19 vaccination series who are 1) aged ≥65 years, 2) aged ≥18 years and reside in long-term care settings, or 3) aged 50–64 years with certain underlying medical conditions^¶^ should receive a COVID-19 vaccine booster dose ≥6 months after completion of the primary vaccination series. In addition, persons aged 18–49 years with certain underlying medical conditions and those aged 18–64 years who are at increased risk for occupational or institutional exposure to SARS-CoV-2 may receive a booster dose based on their individual benefits and risks. Recipients of Janssen COVID-19 vaccine aged ≥18 years should receive a COVID-19 vaccine booster dose ≥2 months after primary vaccination. Any approved or authorized COVID-19 vaccine may be used for the booster dose, regardless of vaccine received for primary vaccination (Box 2). For Pfizer-BioNTech and Janssen, the dose and volume are the same for primary and booster vaccination; for Moderna, the dose and volume of the booster dose (50 *μ*g; 0.25 ml) are one half that used for the primary series (100 *μ*g; 0.5 ml) (Table). As of October 28, 2021, more than 191 million persons in the United States have been fully vaccinated against COVID-19, and more than 15 million have received an additional or booster dose.**

BOX 2 Interim Advisory Committee on Immunization Practices’ recommendations for the use of an additional primary* or booster^†^ dose of COVID-19 vaccines — United States, October 2021^§^

**Additional primary mRNA COVID-19 vaccine dose in immunocompromised persons**

Moderately to severely immunocompromised persons^¶^ aged ≥12 years (Pfizer-BioNTech) or ≥18 years (Moderna) who completed an initial mRNA COVID-19 vaccine series should receive an additional primary mRNA vaccine dose ≥28 days after their second dose.** This recommendation does not apply to immunocompromised recipients of Janssen (Johnson & Johnson) COVID-19 vaccine.

**COVID-19 vaccine booster dose (including heterologous [mix-and-match] booster vaccination)**

**mRNA COVID-19 vaccine (Pfizer-BioNTech, Moderna) recipients**

The following recipients of an mRNA COVID-19 vaccine (Pfizer-BioNTech or Moderna) primary series should receive a single COVID-19 vaccine booster dose ≥6 months after completion of the primary series:

- Persons aged ≥65 years
- Residents aged ≥18 years in long-term care settings
- Persons aged 50–64 years with certain underlying medical conditions**
  The following recipients of an mRNA primary series may receive a COVID-19 vaccine booster dose ≥6 months after completing the primary series based on their individual benefits and risks:
- Persons aged 18–49 years with certain underlying medical conditions**
- Persons aged 18–64 years at increased risk for SARS-CoV-2 exposure and transmission because of occupational or institutional setting

Any FDA-approved or authorized COVID-19 vaccine (Pfizer-BioNTech, Moderna, or Janssen) can be used as the booster dose, at an interval of ≥6 months since primary vaccination.

**Janssen COVID-19 vaccine recipients**
  - Persons aged ≥18 years who received primary vaccination with Janssen COVID-19 vaccine should receive a single COVID-19 vaccine booster dose ≥2 months later.

Any FDA-approved or -authorized COVID-19 vaccine (Pfizer-BioNTech, Moderna, or Janssen) can be used as the booster dose, at an interval of ≥2 months after the primary Janssen vaccine dose.

* Additional primary dose refers to a dose of vaccine administered to persons who likely did not mount a protective immune response after initial vaccination.

^†^ Booster dose refers to a dose of vaccine administered to enhance or restore protection by the primary vaccination, which might have waned over time.

^§^ Additional information is available at https://www.cdc.gov/vaccines/covid-19/clinical-considerations/covid-19-vaccines-us.html.

^¶^ As of October 29, 2021, moderately to severely immunocompromised persons include (but are not limited to) persons with the following conditions or who receive the following treatments: 1) active treatment for solid tumor and hematologic malignancies; 2) receipt of solid-organ transplant and taking immunosuppressive therapy; 3) receipt of chimeric antigen receptor T-cell or hematopoietic cell transplant; 4) moderate or severe primary immunodeficiency; 5) advanced or untreated HIV infection; 6) active treatment with high-dose corticosteroids, alkylating agents, antimetabolites, transplant-related immunosuppressive drugs, cancer chemotherapeutic agents classified as severely immunosuppressive, tumor necrosis factor blockers, and other immunosuppressive or immunomodulatory biologic agents.

** CDC considers persons with certain underlying medical conditions to be at increased risk for developing severe outcomes of COVID-19. An-up-to date list, and supporting evidence, is available at https://www.cdc.gov/coronavirus/2019-ncov/hcp/clinical-care/underlyingconditions.html. As of October 14, 2021, the list of underlying medical conditions includes: asthma, cancer, cerebrovascular disease, chronic kidney disease, certain types of chronic lung diseases, certain types of chronic liver disease, cystic fibrosis, diabetes mellitus (type 1 and type 2), Down syndrome, heart conditions, HIV, hypertension, immune deficiencies, certain mental health disorders (i.e., mood disorders, schizophrenia spectrum disorders), obesity (BMI ≥30 kg/m^2^) and overweight (BMI ≥25 kg/m^2^, but <30 kg/m^2^), pregnancy and recent pregnancy, sickle cell disease, smoking (current and former), solid organ or blood stem cell transplantation, substance use disorders, thalassemia, tuberculosis, and use of corticosteroids or other immunosuppressive medications. A person with a condition that is not listed might still be at greater risk for severe illness from COVID-19 than persons of similar age who do not have the condition and should talk with their health care provider. This list is not exhaustive and should not be used to exclude persons with underlying conditions from recommended preventive measures, including booster doses of COVID-19 vaccines.

Since June 2020, ACIP has convened 20 public meetings to review data relevant to the potential use of COVID-19 vaccines.^††^ To assess the certainty of evidence for benefits and harms of a booster dose, ACIP used the Grading of Recommendations, Assessment, Development and Evaluation (GRADE) approach.^§§^ To further guide its deliberations around the use of an additional or booster dose, ACIP used the Evidence to Recommendations (EtR) Framework to evaluate other factors, including the importance of COVID-19 as a public health problem as well as matters of resource use, benefits and harms, patients’ values and preferences, acceptability, feasibility, and equity for use of the vaccines.^¶¶^

ACIP recommendations for an additional dose of mRNA COVID-19 vaccine in certain immunocompromised persons were guided by data on reduced immunogenicity and effectiveness of the initial primary COVID-19 vaccination series in this population, as well as evidence of an immune response and an acceptable safety profile after an additional mRNA COVID-19 vaccine dose. During the period preceding the emergence of the highly transmissible SARS-CoV-2 B.1.617.2 (Delta) variant, vaccine effectiveness (VE) of a primary mRNA COVID-19 vaccination series against SARS-CoV-2 infection in persons aged ≥16 years was estimated to be 71% (95% confidence interval [CI] = 37%–87%) in immunocompromised persons versus 90% (95% CI = 83%–96%) in the general population and 59% (95% CI = 12%–81%) against COVID-19–associated hospitalization in immunocompromised persons aged ≥18 years versus 91% (95% CI = 86%–95%) in persons who were not immunocompromised (*3*). In a series of small studies, 16% to 80% of solid organ transplant recipients and hemodialysis patients had no detectable antibody response after the second dose of an mRNA COVID-19 vaccine; among these persons, 33% to 55% developed antibodies after receiving an additional dose (*3*). Local and systemic reactions reported after an additional dose of mRNA COVID-19 vaccine in certain immunocompromised persons were mostly mild to moderate and similar to those observed after previous doses; no severe adverse events were reported (*3*). Data were not available to assess immunogenicity or safety of an additional dose in immunocompromised recipients of Janssen COVID-19 vaccine.

To help determine the need for a booster dose in certain populations, ACIP reviewed data on the effectiveness of COVID-19 vaccines after a primary series. In the context of waning vaccine-induced immunity and emergence of the Delta variant in the United States, declines in VE of a primary mRNA COVID-19 vaccination series against SARS-CoV-2 infection have been observed, including among groups recommended to receive early vaccine doses: VE was 75% (95% CI = 60%–85%) to 84% (95% CI = 83%–86%) among adults aged ≥65 years, 53% (95% CI = 49%–57%) among residents of long-term care facilities, and 66% (95% CI = 26%–84%) among health care personnel and other frontline workers during periods of Delta variant predominance (*4,5*). VE of a primary mRNA COVID-19 vaccination series against COVID-19–associated hospitalization overall remains high (78% [95% CI = 62%–87%] to 100% [95% CI = 96%–100%]), although some studies show a slightly lower VE against hospitalization in older adults. Although data are limited, some studies suggest stable VE of Janssen vaccine over time; however, VE of the Janssen vaccine is 58% (95% CI = 12%–80%) to 83% (95% CI = 61%–93%) against SARS-CoV-2 infection and 60% (95% CI = 31%–77%) to 83% (95% CI = 61%–93%) against COVID-19–associated hospitalization among persons aged ≥18 years, which is lower than the estimates reported for mRNA vaccines in most studies (*5*).

ACIP recommendations for a COVID-19 vaccine booster dose in certain persons who had completed primary vaccination were guided by data on immunogenicity, efficacy, and effectiveness of COVID-19 vaccines after booster vaccination, and a review of safety data after COVID-19 vaccine booster doses. Compared with 1 month after the last dose in the primary series, geometric mean ratios of neutralization titers were 1.8 to 3.3-fold higher 1 month after a homologous mRNA COVID-19 vaccine booster dose administered 6 months after completing the primary series, and spike binding antibody titers were 4.6 to 12-fold higher after a homologous Janssen COVID-19 booster dose administered 2–6 months after completing primary vaccination (*6*,*7*). In a small phase I/II clinical trial, both homologous and heterologous (mix-and-match) booster dose administration, in which participants received either a Pfizer-BioNTech, Moderna, or Janssen COVID-19 vaccine primary series followed by a booster dose of the same or different vaccine, resulted in anamnestic immune responses; neutralizing antibody titers after a heterologous booster dose were similar to or higher than those observed after homologous booster vaccination (*6*). Observational studies from Israel demonstrated that the short-term incremental VE of a Pfizer-BioNTech COVID-19 primary series plus booster dose (administered ≥5 months after the second dose) compared with 2 doses, ranged from 70% (95% CI = 62%–76%) in persons aged ≥40 years to 91% (95% CI = 90%–92%) in persons aged ≥60 years (*8*). In placebo-controlled clinical trials, overall efficacy of the Janssen vaccine against moderate to severe COVID-19 ≥14 days after vaccination was 75% (95% CI = 55%–87%) for 2 doses administered 2 months apart versus 53% (95% CI = 47%–58%) for a single dose; in the U.S. study population, efficacy was 94% (95% CI = 59%–100%) after 2 doses and 70% (61%–77%) after 1 dose (6).

In clinical trials for mRNA and Janssen COVID-19 vaccine booster doses, rates of local or systemic adverse events were similar or lower after a booster dose (whether homologous or heterologous) than after the last primary series dose. No serious adverse events related to the vaccine were reported for mRNA COVID-19 vaccine booster doses; for Janssen, three serious adverse events (facial paresis, pulmonary embolism, and cerebrovascular accident) were attributed by the site investigators to booster vaccination within 6 months of administration, among 5,070 booster recipients in the evaluable population (*6,**7*). Outside of clinical trials, more than 13 million persons in the United States had received an additional or booster dose of a COVID-19 vaccine as of October 25, 2021 (predominantly with Pfizer-BioNTech), and no unexpected patterns of adverse events have been observed in national safety surveillance systems (*6*).

From the GRADE evidence assessment, the level of certainty for all benefits and harms of a Pfizer-BioNTech, Moderna, or Janssen COVID-19 vaccine booster dose was type 4 (very low certainty) for the prevention of symptomatic COVID-19, prevention of hospitalization attributable to COVID-19 (Pfizer-BioNTech and Janssen), prevention of death attributable to COVID-19 (Janssen), serious adverse events, and reactogenicity (*6*,*7*). No data were available to assess the GRADE benefit of prevention of SARS-CoV-2 transmission. The main reasons for the low level of certainty in the evidence assessment include small study sizes, lack of a randomized primary series comparison group, short duration of follow-up, and use of immunobridging to infer vaccine efficacy (mRNA vaccines). The GRADE evidence profiles, which provide details on methods for identifying and assessing the supporting evidence, are available at https://www.cdc.gov/vaccines/acip/recs/grade/covid-19-booster-doses.html.

ACIP concluded that the evidence reviewed, including data and considerations from the EtR Frameworks, supported the use of an additional primary dose of an mRNA COVID-19 vaccine for certain immunocompromised recipients of an initial mRNA series, a COVID-19 vaccine booster dose for certain recipients of an mRNA primary series who are at increased risk for exposure to or serious complications of COVID-19, and a COVID-19 vaccine booster dose for all recipients of a Janssen COVID-19 vaccine dose. Additional supporting evidence for the EtR is available at https://www.cdc.gov/vaccines/acip/recs/grade/covid-19-immunocompromised-etr.html and https://www.cdc.gov/vaccines/acip/recs/grade/covid-19-booster-doses-etr.html.

In its deliberations, ACIP discussed the rationale for two different categories of booster dose recommendations among recipients of an mRNA primary series. Persons belonging to groups that ACIP recommends should be vaccinated with a booster dose (Box 2) are groups that are at highest risk for severe COVID-19; several studies suggest waning of VE against hospitalization in older adults. In groups that ACIP recommends may be vaccinated with a booster dose based on individual benefits and risks, evidence suggests that although VE against hospitalization remains high, waning of VE against SARS-CoV-2 infection has been observed. At the September 22–23, 2021, meeting, when booster dose deliberations were limited to Pfizer-BioNTech COVID-19 vaccine, ACIP initially recommended against booster vaccination for persons with frequent occupational or institutional exposure to SARS-CoV-2, given that protection against severe disease in the overall population remains high. However, CDC recommended that persons in this group may receive a booster dose based on their individual benefits and risks, given the implications of waning immunity against infection on health care personnel and other frontline workers, or in settings where the ability to maintain physical distancing or isolation of persons with COVID-19 is more challenging, such as correctional or detention facilities.***^,^^†††^During the October meeting when booster dose deliberations expanded to Moderna and Janssen vaccines, ACIP voted to recommend a COVID-19 vaccine booster dose to recipients of an mRNA primary series (including those who had received Pfizer-BioNTech) who were currently in the risk groups recommended by CDC to receive booster vaccination, including those at occupational or institutional risk for exposure based on individual benefits and risks. This recommendation supersedes the previous recommendations issued by ACIP and CDC in September. Additional information on individual benefit-risk assessments for mRNA booster vaccination is available at https://www.cdc.gov/vaccines/covid-19/clinical-considerations/covid-19-vaccines-us.html. Regarding Janssen COVID-19 vaccine, ACIP discussed the importance of optimizing vaccine-induced protection against SARS-CoV-2 in all recipients of primary vaccination because although VE against infection and hospitalization appears stable over time, VE estimates for Janssen vaccine are overall lower than those observed for mRNA vaccines.

ACIP also emphasized that achieving high and equitable coverage with a COVID-19 primary vaccination series remains the highest priority and is fundamental to reducing COVID-19–related morbidity and mortality. ACIP also stressed the importance of ensuring global equity in access to COVID-19 vaccines for the prevention of disease in vulnerable persons and mitigation of the emergence of SARS-CoV-2 variants.

Before vaccination, providers should provide the EUA Fact Sheet for the vaccine being administered and counsel vaccine recipients about expected systemic and local reactogenicity. Additional clinical education materials are available at https://www.cdc.gov/vaccines/covid-19/index.html, including additional clinical considerations at https://www.cdc.gov/vaccines/covid-19/clinical-considerations/covid-19-vaccines-us.html. The interim recommendations and clinical considerations are based on use of an additional or booster dose of COVID-19 vaccine under an EUA and might change as more evidence becomes available.

## Reporting of Vaccine Adverse Events

FDA requires that immunization providers report vaccine administration errors, serious adverse events, cases of multisystem inflammatory syndrome, and cases of COVID-19 that result in hospitalization or death after administration of COVID-19 vaccine under an EUA (*2*). Adverse events that occur after receipt of any COVID-19 vaccine should be reported to the Vaccine Adverse Events Reporting System (VAERS, https://vaers.hhs.gov or 1-800-822-7967). Any person who administers or receives a COVID-19 vaccine is encouraged to report any clinically significant adverse event, whether or not it is clear that a vaccine caused the adverse event. In addition, CDC has developed a new, voluntary smartphone-based online tool (v-safe) that uses text messaging and online surveys to provide near real-time health check-ins after receipt of a COVID-19 vaccine (https://www.cdc.gov/vsafe).

**Summary**

**What is already known about this topic?**

In the United States, three COVID-19 vaccines are approved or authorized for primary vaccination against COVID-19.

**What is added by this report?**

The Advisory Committee on Immunization Practices issued recommendations for an additional primary mRNA COVID-19 vaccine dose for immunocompromised persons and a COVID-19 vaccine booster dose in eligible groups.

**What are the implications for public health practice?**

Health care professionals play a critical role in COVID-19 vaccination efforts, including for primary, additional primary, and booster vaccination, particularly to protect patients who are at increased risk for severe illness and death.

## Footnotes

* On August 13, 2021, ACIP voted 11–0 in favor of the interim recommendation for use of an additional homologous primary dose of Pfizer-BioNTech (in persons aged ≥12 years) or Moderna (≥18 years) COVID-19 vaccine after an initial series in moderately to severely immunocompromised persons under FDA’s EUA.

† On September 23, 2021, ACIP voted in favor of the following interim recommendations: 1) 15 (in favor)–0 (against) for use of a single Pfizer-BioNTech COVID-19 vaccine booster dose for persons aged ≥65 years and persons aged ≥18 years who reside in long-term care settings, ≥6 months after completion of a Pfizer-BioNTech COVID-19 primary series under FDA’s EUA; 2) 13–2 for use of a booster dose in persons aged 50–64 years with underlying medical conditions ≥6 months after completion of a Pfizer-BioNTech COVID-19 vaccine series; 3) 9–6 for use of a single Pfizer-BioNTech COVID-19 vaccine booster dose, based on individual benefits and risks, for persons aged 18–49 years with underlying medical conditions, ≥6 months after completion of a Pfizer-BioNTech COVID-19 vaccine primary series under FDA’s EUA. ACIP voted 9–6 against an interim recommendation for use of a single Pfizer-BioNTech COVID-19 vaccine booster dose, based on individual benefits and risks, for persons aged 18–64 years who are in an occupational or institutional setting where the impact of COVID-19 infection and transmission are high, ≥6 months after completion of a Pfizer-BioNTech COVID-19 vaccine primary series under FDA’s EUA. The CDC Director accepted ACIP’s recommendations 1, 2, and 3 but did not approve the committee’s recommendation against an interim recommendation for use of a single Pfizer-BioNTech COVID-19 vaccine booster dose, based on individual risks and benefits and made a CDC recommendation that persons aged 18–64 years at high risk for COVID-19 exposure and transmission because of occupational or institutional setting may receive a Pfizer-BioNTech COVID-19 vaccine booster dose ≥6 months after completion of a Pfizer-BioNTech COVID-19 vaccine primary series under FDA’s EUA.

§ On October 21, 2021, ACIP voted in favor of the following interim recommendations: 1) 15–0 for use of a single COVID-19 vaccine booster dose ≥6 months after completion of an mRNA primary series, in the same risk groups for whom CDC recommended a booster dose of Pfizer-BioNTech COVID-19 vaccine, under the FDA’s EUA; and 2) 15–0 for use of a single COVID-19 vaccine booster for persons aged ≥18 years, ≥2 months after receipt of the initial Janssen COVID-19 vaccine dose, under the FDA’s EUA. ACIP recommends that any of the FDA-approved or authorized COVID-19 vaccines (Pfizer-BioNTech, Moderna, or Janssen) can be used for booster vaccination, regardless of the vaccine product used for primary vaccination.

¶ CDC considers persons with certain underlying medical conditions to be at increased risk for developing severe outcomes of COVID-19. An-up-to date list, and supporting evidence, is available at https://www.cdc.gov/coronavirus/2019-ncov/hcp/clinical-care/underlyingconditions.html. As of October 14, 2021, the list of underlying medical conditions includes: asthma, cancer, cerebrovascular disease, chronic kidney disease, certain types of chronic lung diseases, certain types of chronic liver disease, cystic fibrosis, diabetes mellitus (type 1 and type 2), Down syndrome, heart conditions, HIV, hypertension, immune deficiencies, certain mental health disorders (i.e., mood disorders, schizophrenia spectrum disorders), obesity (BMI ≥30 kg/m^2^) and overweight (BMI ≥25 kg/m^2^, but <30 kg/m^2^), pregnancy and recent pregnancy, sickle cell disease, smoking (current and former), solid organ or blood stem cell transplantation, substance use disorders, thalassemia, tuberculosis, and use of corticosteroids or other immunosuppressive medications. A person with a condition that is not listed might still be at greater risk for severe illness from COVID-19 than persons of similar age who do not have the condition and should talk with their health care provider. This list is not exhaustive and should not be used to exclude persons with underlying conditions from recommended preventive measures, including booster doses of COVID-19 vaccines.

** The number of persons who received an additional or booster dose includes any person who is fully vaccinated and has received another dose of COVID-19 vaccine since August 13, 2021. https://covid.cdc.gov/covid-data-tracker/#vaccinations. A person is considered fully vaccinated against COVID-19 ≥2 weeks after receipt of the second dose in a 2-dose series (Pfizer-BioNTech and Moderna) or ≥2 weeks after receipt of the single dose of the Janssen vaccine. As of October 29, 2021, administration of an additional primary dose or a booster dose is not required to be considered fully vaccinated for public health purposes. Additional information can be found at https://www.cdc.gov/coronavirus/2019-ncov/vaccines/fully-vaccinated-guidance.html.

††
https://www.cdc.gov/vaccines/hcp/acip-recs/vacc-specific/covid-19.html

§§ As part of GRADE, the ACIP COVID-19 Vaccines Work Group conducted a systematic review of published and unpublished data. Certainty of evidence was rated on a scale of 1 (high certainty) to 4 (very low certainty). Because of limited available data, GRADE was not used in the assessment of the benefits and harms of an additional primary dose in immunocompromised persons. Additional information on the GRADE approach used by ACIP is available at https://www.cdc.gov/vaccines/acip/recs/grade/about-grade.html.

¶¶
https://www.cdc.gov/vaccines/acip/recs/grade/downloads/acip-evidence-recs-framework.pdf

***
https://www.cdc.gov/coronavirus/2019-ncov/need-extra-precautions/index.html

†††
https://www.cdc.gov/media/releases/2021/p0924-booster-recommendations-.html
